# The association between serum ferritin levels and malignant intraductal papillary mucinous neoplasms

**DOI:** 10.1186/s12885-021-08986-z

**Published:** 2021-11-20

**Authors:** Xiaoling Zhuge, Hao Zhou, Liming Chen, Hui Chen, Xiao Chen, Chuangen Guo

**Affiliations:** 1 Department of Laboratory Medicine, the First Affiliated Hospital, Zhejiang University School of Medicine, 79 Qingchun road, Hangzhou, 310003 China; 2 Department of Radiology, the Affiliated Hospital of Nanjing University of Chinese Medicine, 155 Hanzhong road, Nanjing, 210029 China; 3 Department of Radiology, the First Affiliated Hospital, Zhejiang University School of Medicine, 79 Qingchun road, Hangzhou, 310003 China

**Keywords:** Intraductal papillary mucinous neoplasms, Ferritin, Malignancy, Invasive carcinoma

## Abstract

**Background:**

Serum ferritin levels are elevated in many malignancies. In this study, we showed the performance of serum ferritin in identifying malignant intraductal papillary mucinous neoplasms (IPMNs).

**Methods:**

A total of 151 patients with pathologically confirmed IPMNs were enrolled. Serum tumor biomarker (carbohydrate antigen 19–9 (CA19–9) and carcinoembryonic antigen (CEA)) levels and serum ferritin levels were recorded. Lesion location, tumor size, diameter of the main pancreatic duct (MPD), mural nodule, and IPMN type, were collected from imaging examinations. IPMNs with high grade dysplasia and associated invasive carcinoma were considered malignant IPMNs.

**Results:**

Serum ferritin levels in patients with malignant IPMNs were higher than those in patients with nonmalignant IPMNs (*p* <  0.05). Serum ferritin was an independent factor for the occurrence of malignant IPMNs (odds ratio (OR) = 1.18, 95% confidence interval (CI):1.01–1.39). A similar trend was found between high serum ferritin (> 149 ng/ml) and malignant IPMNs (OR = 5.64, 95% CI:1.78–17.92). The area under the curve (AUC) of serum ferritin was higher than that of CEA and CA19–9 in identifying malignant IPMNs (AUC = 0.67 vs. AUC = 0.58, 0.65). The combination of serum ferritin with IPMN type showed a similar performance to MPD diameter and the combination of serum CA19–9 with IPMN types in identifying malignant IPMNs (AUC = 0.78 vs. AUC = 0.79, 0.77) and invasive carcinoma (AUC = 0.77 vs. AUC = 0.79, 0.79).

**Conclusions:**

Elevated serum ferritin is a factor associated with malignant IPMNs. Serum ferritin may be a useful marker for identifying malignancy in IPMNs.

## Introduction

Intraductal papillary mucinous neoplasms (IPMNs) represent one type of cystic pancreatic neoplasms. The critical issue of IPMNs is their potential for malignant transformation. More than 40% of IPMNs are high-grade dysplasia or invasive carcinoma [1]. IPMNs are divided into three types based on the site of origin in the pancreatic ductal system: main-duct (MD) type, side-branch duct (BD) type, and mixed type (MT). MD-IPMNs are less common but have a higher risk of malignancy than BD-IPMNs [2]. A total of 38–87% of MD-IPMNs had malignant histological features in resected specimens [3, 4]. Surgical resection is usually performed for MD-IPMNs and MT-IPMNs due to their high risk of malignancy [5]. In 2012, the threshold diameter of the main pancreatic duct (MPD) for MD-IPMNs was set at 5.0 mm [6]. The need for surgical resection is not only based on MPD diameter but also based on “high-risk stigmata” and “worrisome features”, such as biliary obstruction and the presence of mural nodules. The early detection of the malignancy in IPMNs is important for treatment planning [7]. Whether other potential high-risk factors exist for identifying malignant IPMNs needs to be further explored.

Tumor biomarkers are usually elevated in malignant IPMNs [8, 9]. Hirono et al. showed that carcinoembryonic antigen (CEA) in the pancreatic juice was a biomarker of carcinomas in MD- and MT-IPMNs [8]. In addition, they also found that serum carbohydrate antigen 19–9 (CA19–9) was independently related to malignancy in MT-IPMNs [7]. Similar results were also reported in other studies [10–14]. Previous guidelines have not reported other potential serum biomarkers in the management of IPMNs.

Ferritin, consisting of 24 subunits, is ubiquitously expressed as an iron storage protein. The function of serum (extracellular) ferritin in human biology is not completely understood [14]. Elevated serum ferritin is found in chronic and acute inflammation [15]. Moreover, serum ferritin levels are elevated in many malignancies [15], including hepatocellular carcinoma, hematological malignancies, and lung and breast cancer. Several studies also showed that elevated serum ferritin can be found in patients with pancreatic duct adenocarcinoma [16, 17]. Serum ferritin is also related to short survival in patients with T-cell lymphoma [18], hepatocellular carcinoma [19, 20] and pancreatic cancer [21, 22]. Ferritin may also be associated with tumor progression [23]. However, the role of serum ferritin in IPMNs is poorly understood. Therefore, in this study, we showed the associations between serum ferritin and malignant IPMNs. In addition, we also showed the performance of serum ferritin in identifying the malignant IPMNs.

## Materials and methods

### Patients

A total of 151 patients with histologically proven IPMNs who underwent surgery during 2011–2020 were included. The patients collection protocol was described in an our previous study [24]. Briefly, the patients’ demographic information, medical history, clinical data, preoperative symptoms, serum CA19–9 level, serum CEA levels, and serum ferritin levels, and pathological data were collected from medical records. Serum ferritin, CA19–9 and CEA levels were determined within 1 week before operation. Diabetes mellitus (DM) was diagnosed based on plasma glucose levels or DM history. None of the patients received endoscopic ultrasound examinations. This study was approved by the Institutional Review Board of the the First Affiliated Hospital, Zhejiang University School of Medicine, and the requirement for informed consent was waived because of the retrospective design. The study adhered to the Declaration of Helsinki.

### Imaging data

The following radiological data were collected from computed tomography (CT) or magnetic resonance imaging (MRI): lesion location (head-neck or body-tail), tumor size, MPD diameter, and mural nodule. If the lesion was too large, the location was evaluated based on the center of the cyst. If there were multiple lesions, the location of the main cyst was judged. IPMNs were classified into three types based on the degree of involvement of the pancreatic ductal system: MD-IPMNs, BD-IPMNs, and MT-IPMNs. MD-IPMNs were considered when there was segmental or diffuse involvement of the MPD; BD-IPMNs were considered when the lesions communicated with the MPD.

### Histological examinations

The histological evaluation of each IPMN was based on the World Health Organization guidelines for IPMNs. The IPMNs were divided into three grades based on cytoarchitectural atypia: low-intermediate dysplasia, high-grade dysplasia, and invasive adenocarcinoma. Those IPMNs with high-grade dysplasia and associated invasive carcinoma were considered as malignant ones. Lymph node metastasis (yes vs. no) and peripancreatic extension (organ invasion and vascular invasion) were also evaluated.

### Statistical analysis

The data was shown as as mean ± standard deviation (continuous data) or was shown as number (qualitative data). Clinicopathological variables including continuous data, such as patient’s age, tumor sizes, the serum levels of CEA, CA19–9, ferritin, MPD diameter were compared by Independent-sample T test or Mann-Whitney U-test. Qualitative data like sex, dysplasia level, tumor type (MD, BD and MT), tumor location (Head-neck vs Body and Tail), whether accompanied with chronic cholecystitis, pancreatitis, abdominal symptoms, diabetes, lymph node metastasis, peripancreatic extension, mural nodule were compared by Chi-square test or Fisher’s exact test. Univariable and multivariable logistic regression analyses were adopted to identify the associated factors for malignant IPMNs and invasive carcinomas. Additional adjustment with diabetes, pancreatitis and chronic cholecystitis were also performed because they may be related to serum ferritin levels. Receiver operating characteristic (ROC) curves were used to evaluate the performance of serum ferritin, MPD diameter, CA19–9, CEA in identifying malignant IPMNs and invasive carcinoma. *P* <  0.05 was considered as statistically significant.

## Results

### Patient clinicopathological features of malignant and non-malignant IPMNs

The clinical data is shown in Table 1. Overall, 47 patients (31.1%) had malignant IPMNs. Most nonmalignant IPMNs were BD-IPMNs; in contrast, malignant IPMNs was commonly seen among MD-IPMNs. The MPD diameter in malignant IPMNs was significantly larger than that in nonmalignant IPMNs (*p* <  0.05). Patients with malignant IPMNs had higher level of serum CA19–9 and serum ferritin than those with nonmalignant IPMNs. Peripancreatic extension and mural nodules were more commonly seen in malignant IPMNs (*p* <  0.01).

**Table 1:** Clinical data in malignant and nonmalignant IPMNs

	Total (*n* = 151)	Malignant IPMNs (*n* = 47)	Nonmalignant IPMNs (*n* = 104)	p
Age	63.28 ± 9.47	63.59 ± 8.99	62.60 ± 10.50	0.55
Size	3.69 ± 1.98	3.93 ± 1.63	3.58 ± 2.11	0.34
Sex (male/female)	84/47	29/11	55/36	0.28
Dysplasia				
Low-intermediate grade	104	/	104	
High-grade	24	24	0	
Invasion	23	13	0	
Type				< 0.01
Main	24	16	8	
Branch	73	9	64	
Mixed	54	22	32	
Location				0.15
Head-neck	95	34	61	
Body and Tail	56	13	43	
CEA (ng/ml)	3.57 ± 3.61	4.75 ± 5.65	3.03 ± 1.95	0.09
CEA > 5.0	24	10	14	0.22
CA19–9 (U/ml)	48.68 ± 228.65	99.04 ± 396.08	26.19 ± 70.67	0.002
> 37	29	17	12	< 0.001
Ferritin (ng/ml)	275.03 ± 284.65	370.58 ± 358.19	231.84 ± 233.76	0.001
> 232	60	26	34	0.009
MPD diameter	0.61 ± 0.41	0.90 ± 0.49	0.48 ± 0.29	< 0.001
Chronic cholecystitis	56	17	39	0.87
Pancreatitis	3	0	3	0.24
Abdominal Symptoms	67	23	44	0.92
Diabetes	23	9	14	0.13
Lymph node metastasis (yes vs no)	2	2	0	0.09
Peripancreatic extension	5	5	0	0.003
Mural nodule	17	13	4	< 0.01

Malignant IPMNs were defined as those with high grade dysplasia and associated invasive carcinoma

*CA 19–9* carbohydrate antigen 19–9; *CEA* carcinoembryonic antigen; *MPD* main pancreatic duct

### *Patient clinicopathological features of IPMN with and without invasive* carcinoma

Next, we compared clinicopathological features between IPMN patients with and without invasive carcinoma (Table 2). Most of invasive carcinomas were MD type and mixed type (*p* <  0.01). In addition, invasive carcinomas had a larger pancreatic duct diameter than noninvasive carcinomas (*p* <  0.001). In addition, patients with invasive carcinoma had higher serum CEA and CA19–9 levels than patients without invasive carcinoma (*p* <  0.05). Abnormal CA19–9 levels were also more common in IPMN patients with invasive carcinoma than in those without invasive carcinoma. However, no significant difference was found in abnormal CEA levels between those with and without invasive carcinoma. Diabetes, lymph node metastasis, extrapancreatic expansion and the presence of mural nodules were more common in patients with invasive carcinoma (*p* <  0.05).

**Table 2:** Clinical data in IPMN with and without invasive carcinoma

	Invasive carcinoma (*n* = 23)	Noninvasive carcinoma (*n* = 128)	p
Age	61.52 ± 11.40	63.60 ± 9.09	0.51
Size	3.95 ± 1.85	3.64 ± 2.00	0.27
Sex (male/female)	15/8	80/48	0.80
Type			< 0.01
Main-duct	9	15	
Brach-duct	3	70	
Mixed	11	43	
Location			0.24
Head-neck	17	78	
Body and Tail	6	50	
CEA (ng/ml)	7.11 ± 8.05	3.06 ± 1.98	0.006
CEA > 5.0	5	14	0.11
CA19–9 (U/ml)	160.00 ± 568.57	29.40 ± 70.66	0.004
> 37	10	19	0.001
Ferritin (ng/ml)	387.66 ± 465.68	254.79 ± 235.41	0.18
> 232	12	48	0.19
PD diameter	0.98 ± 0.49	0.54 ± 0.36	< 0.001
Chronic cholecystitis	10	46	0.49
Pancreatitis	0	3	1.0
Abdominal Symptoms	9	58	0.58
Diabetes	7	18	0.05
Lymph node metastasis (yes vs no)	2	0	0.02
Extrapancreatic expansion	5	0	< 0.01
Mural nodule	9	8	< 0.01

*CA 19–9* carbohydrate antigen 19–9; *CEA* carcinoembryonic antigen; *MPD* main pancreatic duct

### The association between tumor grade and serum CEA, CA19–9, ferritin level

**Fig. 1:**
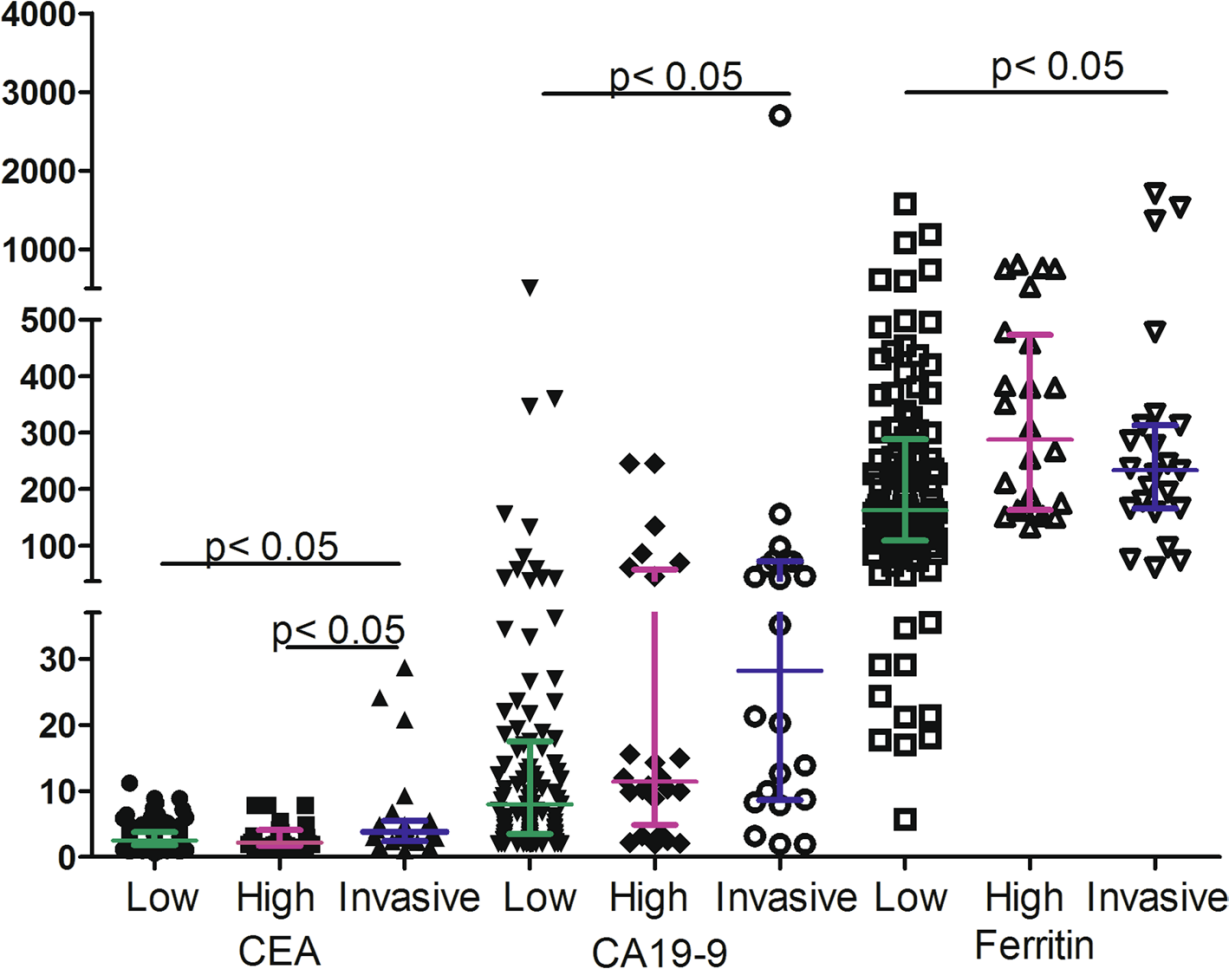
The levels of serum carbohydrate antigen 19–9 (CA19–9) level, serum carcinoembryonic antigen (CEA) levels, and serum ferritin levels in intraductal papillary mucinous neoplasms (IPMNs). IPMNs were divided into low-moderate, high grade dysplasia and associated invasive carcinoma

The serum CEA, CA19–9 and ferritin levels are shown in Fig. 1. Serum CEA, CA19–9 and ferritin levels were related to tumor malignancy (*p* <  0.05). IPMNs with malignant characteristics, including high-grade dysplasia and invasive carcinoma, always had higher serum CEA, CA19–9 and ferritin levels than non-malignant IPMNs (p <  0.05).

### ROC analysis

**Fig. 2:**
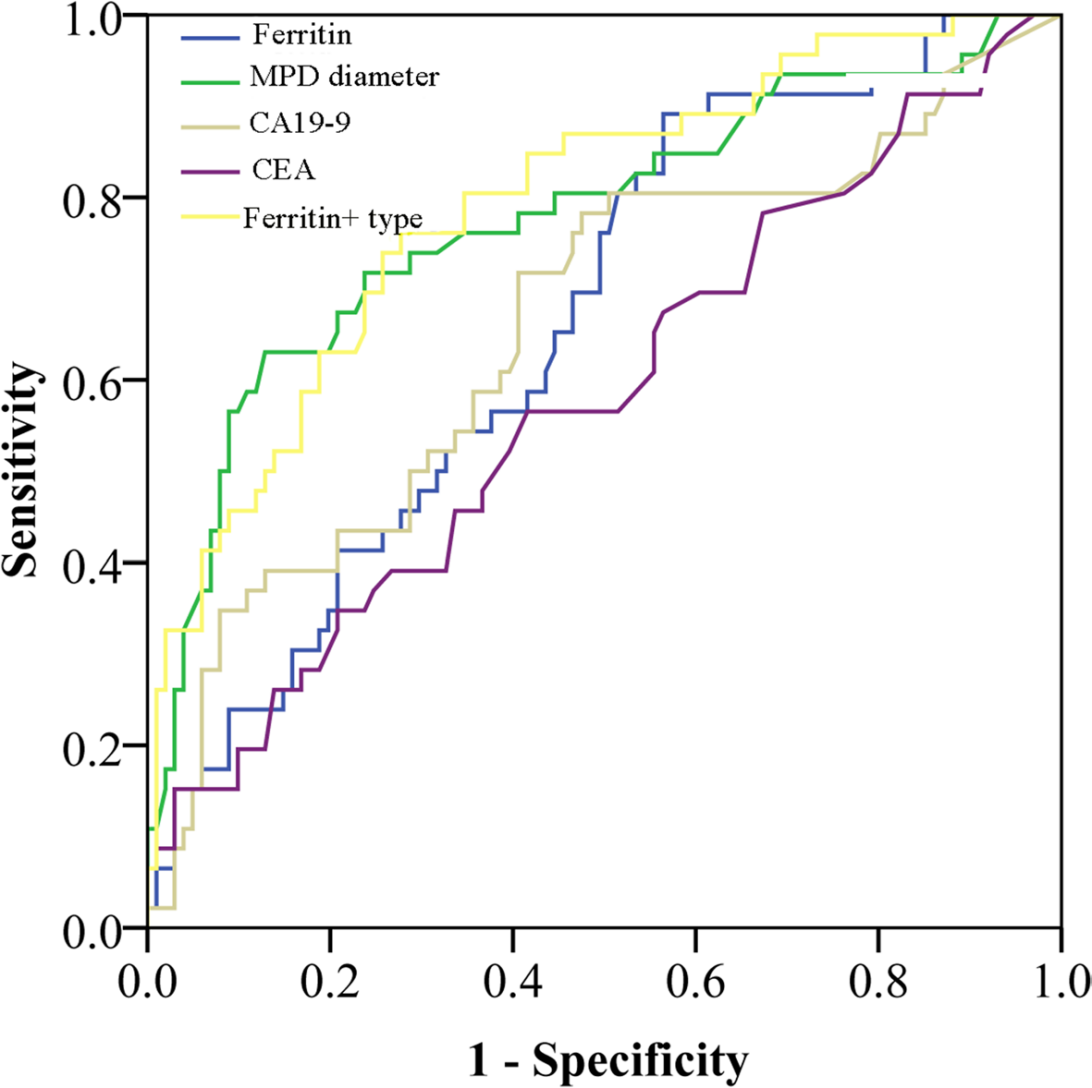
The receiver operating characteristic (ROC) curves of serum carbohydrate antigen 19–9 (CA19–9) level, serum carcinoembryonic antigen (CEA) levels, serum ferritin levels, main pancreatic duct (MPDF) diameter, and combination of serum ferritin with tumor type (ferritin + IPMN type) in predicting malignant intraductal papillary mucinous neoplasm (IPMN) (**A**) and invasive carcinoma (**B**)

The performance of serum CEA, CA19–9, ferritin, pancreatic duct diameter and the combination of ferritin with tumor type in evaluating malignant IPMNs and carcinomas are shown in Fig. 2. When serum ferritin, CA19–9 or CEA was used alone, they had smaller areas under the curve (AUCs) for evaluating malignant IPMNs than MPD diameter (AUC = 0.78, 95% CI: 0.69–0.86) or the combination of ferritin with tumor type (AUC = 0.79, 95% CI: 0.71–0.87) (Fig. 2A). The AUC for identifying malignant IPMNs of serum ferritin was higher than that of CEA and CA19–9 (0.67 vs. 0.58, 0.65). The cutoff value for serum ferritin was 149 ng/ml, with a sensitivity of 89.4% and specificity of 55.4% in identifying malignant IPMNs.

### Associated factors with malignant IPMNs

The associated factors for malignant IPMNs are listed in Table 3. Compared with MD-IPMNs, BD-IPMNs (OR = 0.07, 95% CI: 0.02–0.21) and MT-IPMNs (OR = 0.34, 95% CI: 0.13–0.94) had a lower risk for being malignant IPMNs. This association between BD-IPMNs and malignant IPMNs remained after adjusting for diabetes, pancreatitis and cholecystitis (OR = 0.06, 95% CI: 0.13–0.94). We also found that serum ferritin was an independent factor associated with malignant IPMNs (OR = 1.19, 95% CI: 1.04–1.35), and such trends remained after controlling for confounders, such as diabetes, pancreatitis and cholecystitis. A similar trend was found when serum ferritin was set as a categorical variable (threshold = 149 ng/ml) (OR = 5.64, 95% CI:1.78–17.92). However, age, sex, tumor size and tumor location had no significant association with malignant IPMNs. Serum ferritin levels were not associated with invasive carcinoma in multivariable logistic regression analysis (Table 4).

**Table 3:** Associated factors with malignant IPMNs

	A				B	
variables	Univariable	Multivariable			Multivariable	
		Model 1	Model 2	Model3	Model 1	Model 2
	OR (95%CI)	OR (95%CI)	OR (95%CI)	OR (95%CI)	OR (95%CI)	OR (95%CI)
Age	0.99(0.95–1.03)	0.97(0.93–1.02)	0.98(0.93–1.02)	0.97(0.93–1.02)	0.97(0.93–1.02)	0.97(0.93–1.02)
Sex	0.54(0.26–1.15)	0.57(0.24–1.38)	0.56(0.23–1.37)	0.56(0.23–1.36)	0.84(0.33–2.18)	0.83(0.32–2.15)
Size (cm)	1.09(0.91–1.30)	1.06(0.87–1.29)	1.05(0.86–1.28)	1.04(0.85–1.27)	1.07(0.87–1.31)	1.06(0.87–1.30)
Type						
Main-duct	1	1	1	1	1	1
Branch-duct	0.07(0.02–0.21)	0.06 (0.02–0.21)	0.06 (0.02–0.20)	0.06 (0.02–0.21)	0.08 (0.02–0.27)	0.08 (0.02–0.28)
Mixed	0.34(0.13–0.94)	0.36(0.12–1.07)	0.34(0.11–1.04)	0.35(0.12–1.08)	0.39(0.13–1.24)	0.41(0.13–1.28)
Location						
Head-neck	1	1	1	1	1	1
Body-Tail	0.54(0.26–1.15)	0.61(0.24–1.52)	0.51(0.19–1.37)	0.53(0.19–1.43)	0.44(0.16–1.21)	0.47(0.17–1.28)
Ferritin (×100, ng/ml) Ferritin (> 149 vs ≤ 149 ng/ml)	1.19(1.04–1.35)	1.19(1.02–1.39)	1.19(1.02–1.39)	1.18(1.01–1.39)	5.70(1.80–18.11)	5.64(1.78–17.92)

A: Serum ferritin was continuous variable; Model 2 was adjusted with pancreatitis and chronic cholecystitis; Model 3 was additionally adjusted with diabetes

B: Serum ferritin was categorical variable; Model 1 was adjusted with pancreatitis and chronic cholecystitis; Model 2 was additionally adjusted with diabetes

*CI* confidence interval; *OR* odds ratio

**Table 4:** Associated factors with invasive carcinoma

variables	Univariable	Multivariable		
		Model 1	Model 2	Model 3
	OR (95%CI)	OR (95%CI)	OR (95%CI)	OR (95%CI)
Age	0.98(0.94–1.02)	0.97(0.92–1.02)	0.97(0.92–1.03)	0.97(0.92–1.03)
Sex	0.89(0.37–2.74)	1.17(0.42–3.29)	1.17(0.42–3.29)	1.17(0.41–3.32)
Size (cm)	1.15(0.35–2.25)	0.99(0.76–1.29)	0.99(0.76–1.29)	0.93(0.70–1.24)
Type				
Main-duct	1	1	1	1
Branch-duct	0.07(0.02–0.30)	0.06 (0.01–0.26)	0.06 (0.01–0.26)	0.06 (0.01–0.27)
mixed	0.43(0.15–1.23)	0.37(0.12–1.18)	0.37(0.12–1.19)	0.39(0.12–1.26)
Location				
Head-neck	1	1	1	1
Body-Tail	0.55(0.20–1.49)	0.57(0.18–1.82)	0.57(0.17–1.92)	0.64(0.19–2.19)
Ferritin (×100, ng/ml)	1.14(1.001–1.29)	1.14 (0.99–1.32)	1.14 (0.98–1.32)	1.11 (0.96–1.30)

Model 2 was adjusted with pancreatitis and chronic cholecystitis

Model 3 was additionally adjusted with diabetes

*CI* confidence interval; *OR* odds ratio

## Discussion

IPMNs are one type of potentially malignant cystic lesion of the pancreas. The identification of malignancy is still a challenge even though several guidelines have shown ““high-risk stigmata“ and “worrisome features” [25]. It is of great importance to determine the risk factors of malignant IPMNs or carcinomas. Our study demonstrated that serum ferritin levels in patients with malignant IPMNs were significantly higher than those in patients with nonmalignant IPMNs. Multivariate logistic analysis also showed that serum ferritin level was an independent factor associated with malignant IPMNs. ROC analysis showed that serum ferritin level had a similar predictive performance to Ca19–9 in identifying malignant IPMNs. Thus, serum ferritin may have potential in predicting malignant IPMNs.

Previous studies have shown that several morphologic features of IPMNs and serum tumor biomarkers were associated with malignant IPMNs and invasive carcinoma [13, 15, 26]. MPD and mural nodules (enhancing solid component) were the two of main high risk stigmata. Our data also showed that MPD diameter and mural nodules were associated with invasive IPMNs which was in accordance with those previous findings. Recently, other possible factors that may be associated with malignant IPMNs have also been reported. Pergolini et al. [27]. pointed out that patients with IPMNs have a higher risk of high-grade dysplasia or invasive cancer when accompanied by diabetes or weight loss. Simpson et al. [28] found that combined pancreatic fluid interleukin-1β with pancreatic fluid prostaglandin E2 and serum CA19–9 could help discriminate high-grade dysplasia/invasive IPMNs from low/moderate-risk IPMNs. Elevated serum ferritin levels can occur in many malignancies [15]. However, few studies have shown the relationship between serum ferritin and malignant IPMNs.

Serum ferritin levels have been widely used as a tumor biomarker for clinical diagnosis and prognosis evaluation [15]. Ferritin may be used as a marker in renal cell carcinoma [29]. Orlandi et al. [30] showed that ferritin light chain had a good capacity for predicting malignant breast lesions (AUC = 0.86). Gao et al. [31] found that the combination of serum ferritin with four other tumor biomarkers had improved sensitivity for diagnosing colorectal cancer compared with CEA alone. Ferritin light chain (FTL) was an independent prognostic marker in patients with node-negative breast cancer tumors (HR:1.30; 95% CI:1.10–1.50) [32]. The possible role of serum ferritin in pancreatic cancer has also been investigated. Kalousová et al. found that elevated serum levels of ferritin indicated poor prognosis in the patients with pancreatic cancer [16]. Alkhateeb et al. also showed a similar result [33]. Pancreatic cancer patients who showed normal serum ferritin levels after chemoradiotherapy had a longer median progression-free survival than patients whose serum ferritin levels were not restored [22]. To our knowledge, no study has shown an association between serum ferritin levels and malignant IPMNs or invasive carcinoma. Our data indicated that serum ferritin levels were associated with malignant IPMNs. ROC analysis showed that the predictive performance of serum ferritin was similar to that of CA19–9, and higher than that of CEA. Elevated CA19–9 has been adopted as a “worrisome feature”. Serum ferritin may be another such feature. Our study is a primary exploration. Further explorations are required.

How ferritin affects the occurrence of malignancy in IPMNs is not clarified. Some studies have shown that the mechanism of tumor transformation and progression induced by ferritin. Ferritin is abundant in circulation and differently overexpressed in various malignant tumor tissues. A study reported that extracellular ferritin might exert immunosuppressive effects on myeloid cells and lymphocytes by modulating iron availability and that H-ferritin (known as heavy chain ferritin, a subunit of ferritin) had suppressive effects on myelopoiesis [15], which led to an immunosuppression in cancer patients. Coffman et al. [34] found that ferritin could promote assembly of tumor endothelial cells, promote angiogenesis during tumor growth and enhance the migration by antagonizing the antiangiogenic effects of HKa (cleaved high molecular weight kininogen). Tumor-associated macrophages (TAMs) in the breast tumor stroma can secrete ferritin which leads to the proliferation of epithelial breast cancer cells) [14]. Thus, regulating ferritin downregulation in TAMs may reduce their ability to maintain iron stores, which can result in tumor cell death. Conklin et al. [35] pointed out that ferritin might decrease the efficacy of chemotherapeutic drugs because of its antioxidant nature. The inhibition of H-ferritin in ferritin-rich glioma cells could sensitize cancer cells to the chemotherapeutic agents [36]. High ferritin expression enhanced progression and increased resistance to oxidative stress in metastatic melanoma [22, 37]. It is not clear which pathways are involved in the occurrence of malignancy in IPMNs. Further in vivo or in vitro studies are needed to show the biological role of ferritin in IPMNs.

Cytological findings are also an important high-risk stigmata in several guidelines. Endoscopic ultrasound-guided fine-needle aspiration (EUS-FNA) has been a routine practice to collecting samples for cytology and cystic fluid analysis [38]. However, the accuracy rate and role of EUS-FNA in the identification of malignancy has not been proven [38]. A recent meta-analysis showed that the accuracy rate, sensitivity, and specificity of EUS-guided through-the-needle biopsy (EUS-TTNB) were 78.8, 82.2, and 96.8%, respectively [38]. The diagnostic yield of EUS-TTNB may be superior to that of EUS-FNA [38, 39]. The pooled concordance of EUS-TTNB with the histological grade of mucinous cysts was 75.6% [39]. The combination of EUS-TTNB and serum ferritin for identifying malignant IPMNs may be worthy of further study.

There are several limitations in our study. First, the number of BD-IPMNs was relatively small. Second, our study was a single-institution study which may cause selection bias, and a multicenter study should be performed to confirm our findings. Third, cyst fluid tumor marker levels [40, 41] and nonetumor biomarkers, such as microRNA and DNA [41, 42], are also useful for the identification of malignant IPMNs. However, we did not determine the ferritin level in cystic fluid or tumor tissues. Fourth, we did not perform a longitudinal study to show the associations between baseline serum ferritin and occurrence of malignant IPMNs. Fourth, some IPMN patients who did not undergo surgical resection were not included in our analysis, such as those patients undergoing surveillance and those who lost the chance to undergo surgery. The loss of these populations may affect the results. However, approximately 40% of our population that underwent surgery did not have “high-risk stigmata” or “worrisome features”. These subjects were comparable to the patients who underwent surveillance. Therefore, our results may also be useful for identifying malignancy in general population with IPMNs. However, further studies are required. Finally, as a retrospective study, we did not evaluate the association between serum ferritin and survival or recurrence after surgery and a prospective study is necessary in the future.

In conclusion, our study reports that elevated serum ferritin is associated with the presence of malignant IPMNs. Moreover, serum ferritin is also associated with invasive carcinoma in MPD-involved IPMNs. Serum ferritin may be a potential biomarker for identifying malignant IPMNs, especially for MPD-involved IPMNs. Multicenter studies and longitudinal studies are required to show the role of serum ferritin in the occurrence of malignant IPMNs.

## Data Availability

All data generated or analyzed during this study are included in this published article (and its Supplementary Information files).

## Abbreviations

AUC: Area under the curve
BD: Branch duct
CA19–9: Carbohydrate antigen 19–9 (CA19–9)
CEA: Serum carcinoembryonic antigen
CT: Computed tomography
DM: Diabetes mellitus
EUS-FNA: Endoscopic ultrasound-guided fine-needle aspiration
EUS-TTNB: EUS-guided through-the-needle biopsy
FTL: Ferritin light chain
IPMN: INTRADUCTAL papillary mucinous neoplasms
MD: Main duct
MPD: Main pancreatic duct
MRI: Magnetic resonance imaging
OR: Odds ratio
ROC: Receiver operating characteristic
TAMs: tumor-associated macrophages
